# Plasma heparan sulfate structural variation and phenotypic heterogeneity in pediatric Acute Respiratory Distress Syndrome

**DOI:** 10.1038/s41598-026-59291-3

**Published:** 2026-06-26

**Authors:** Colin J. Sallee, Aline B. Maddux, Joseph A. Hippensteel, Daniela Markovic, Zhangjie Wang, Jian Liu, Kaori Oshima, Robert P. Richter, Andreas Schwingshackl, Peter M. Mourani, David Elashoff, Eric P. Schmidt, Anil Sapru

**Affiliations:** 1 Department of Pediatrics, Division of Pediatric Critical Care Medicine, David Geffen School of Medicine at University of California Los Angeles and Mattel Childrens Hospital, Los Angeles, CA USA; 2 Department of Pediatrics, Section of Pediatric Critical Care, University of Colorado School of Medicine and Children’s Hospital Colorado, Aurora, CO USA; 3 Department of Medicine, Division of Pulmonary Sciences and Critical Care Medicine, University of Colorado Anschutz Medical Campus, Aurora, CO USA; 4 Departments of Medicine and Computational Biology, David Geffen School of Medicine at University of California Los Angeles, Los Angeles, CA USA; 5 Glycan Therapeutics, Raleigh, NC USA; 6 Division of Chemical Biology and Medicinal Chemistry, Eshelman School of Pharmacy, University of North Carolina at Chapel Hill, Chapel Hill, NC USA; 7 Department of Medicine, Division of Pulmonary and Critical Care Medicine, Harvard Medical School and Massachusetts General Hospital, Boston, MA USA; 8 Department of Pediatrics, Division of Pediatric Critical Care Medicine, University of Alabama at Birmingham Heersink School of Medicine, Birmingham, AL USA; 9 Department of Pediatrics, Division of Pediatric Critical Care Medicine, University of Michigan Medical School, Ann Arbor, MI USA

**Keywords:** Pediatric Acute Respiratory Distress Syndrome, Endothelial glycocalyx, Heparan sulfate, Heparanase, Biological heterogeneity, Mass spectrometry, Biochemistry, Biomarkers, Diseases, Medical research

## Abstract

Endothelial glycocalyx (eGCX) shedding contributes to microvascular endotheliopathy in Acute Respiratory Distress Syndrome (ARDS) and may represent an underrecognized source of phenotypic heterogeneity. We examined whether circulating heparan sulfate (HS) signatures, as readouts of eGCX shedding, capture patterns of inter-patient biological variation distinct from other eGCX components and conventional protein biomarkers, whether specific HS structural features are enriched, and whether these signatures are associated with heparanase-1 (HPSE) activity. We retrospectively analyzed prospectively collected plasma samples (2018–2020) from children with and without pediatric ARDS (PARDS). Plasma levels (ng/mL) of sulfated and non-sulfated HS disaccharides (following enzymatic digestion of total [unfractionated] HS), and HPSE activity (U/mL) were measured using mass spectrometry, while protein biomarkers were assessed by multiplex assay. Among 46 children (36 PARDS, 10 no PARDS), principal component analysis identified three components explaining > 60% of the variance. The primary component (PC1) was characterized by an HS-driven endothelial signature and was distinct from an inflammatory protein signature captured by PC2. In PARDS, children with higher PC1 scores had worse organ dysfunction and fewer ventilator-free days after adjustment for PC2 and PC3. Higher total HS levels were further associated with selective enrichment of sulfated HS motifs, whereas the opposite pattern was observed in non-PARDS. HPSE activity positively correlated with circulating HS levels. These preliminary findings suggest that circulating HS signatures capture a distinct and clinically meaningful dimension of PARDS heterogeneity.

**Supplementary Information:**

The online version contains supplementary material available at https://doi.org/10.1038/s41598-026-59291-3.

## Introduction

Acute respiratory distress syndrome (ARDS) exhibits substantial phenotypic (clinical and biological) heterogeneity that has been postulated to limit the success of therapeutic trials^1^. Accumulating evidence suggests that the extent of systemic inflammation, reflected in circulating cytokines and innate immune activation markers, partitions patients into prognostic and potentially, predictive biological phenotypes^2^. Yet, even with the growing use of biological data to support precision medicine strategies in critical care medicine, current ARDS biological phenotyping frameworks remain limited and likely, incomplete.

Microvascular endotheliopathy is a central mechanism of organ injury in ARDS^3^ and critical illness^4^ more broadly; however, its relative contribution to biological heterogeneity and phenotypic classification in ARDS remains comparatively understudied. An early and key driver of microvascular endotheliopathy is shedding of the endothelial glycocalyx (eGCX)^5^, a heparan sulfate (HS) proteoglycan-rich extracellular matrix that lines the luminal surface of the endothelium and is essential for maintaining endothelial barrier integrity^6^, regulating vascular tone^7^, and preserving inflammatory homeostasis^8^. Disruption of the eGCX by enzymatic “sheddases”^9^, reactive oxygen species^10^ and shear stress^11^ leads to loss of these protective functions. Circulating HS fragments serve not only as mechanistic readouts of eGCX shedding^12,13^ but also display variable sulfation motifs that may actively influence cellular signaling through interactions with protein ligands, growth factors, and cell-surface receptors^14^. Accordingly, these structural features may provide insight into mechanisms driving eGCX shedding and into their broader contributions to ARDS pathophysiology.

In this study of mechanically ventilated children with pediatric ARDS (PARDS), we pursued three complementary objectives: (1) to examine whether circulating eGCX-derived HS signatures capture patterns of inter-patient biological variation distinct from those reflected by other eGCX components and canonical biomarkers of innate immune and endothelial activation; (2) to assess whether specific HS structural features (i.e., sulfation motifs) are selectively enriched in PARDS; and (3) in an exploratory analysis, to evaluate the relationship between HS signatures and heparanase-1 (HPSE) activity, the principal HS-degrading enzyme^15^. We hypothesized that circulating eGCX-derived HS signatures represent a unique molecular signature of endothelial injury and capture clinically meaningful heterogeneity among children with PARDS. We further hypothesized that variation in HS signatures arises not only from differences in overall abundance but also from qualitative differences in sulfation patterns.

## Methods

### Study design

The parent study was conducted from 2018 to 2020 at an urban, quaternary children’s hospital^16^. It was approved by the Colorado Multiple Institutional Review Board (IRB 18–0463) and conducted in accordance with institutional standards and the 1975 Helsinki Declaration. Eligible patients’ parent/guardians were approached to obtain written informed consent for participation.

We conducted a retrospective, secondary analysis of this prospectively enrolled cohort of children (≥ 1 month and ≤ 18 years old) with acute respiratory failure (ARF) requiring invasive mechanical ventilation (IMV) for a minimum of three days. The original study excluded patients who had support limitations, if the investigator or PICU team felt the child was inappropriate to enroll, or if the patient had a pre-existing tracheostomy. Blood was collected on day of consent, which was typically 3–5 days after IMV initiation. Among patients with available blood for analysis, a convenience sample of children with and without PARDS was identified from the parent cohort. PARDS diagnosis was based on the Pediatric Acute Lung Injury Consensus Conference-2 (PALICC-2) criteria^17^. This group was compared to children with ARF receiving IMV for neurologic failure or a non-pulmonary procedure/surgery similar to prior analyses of this cohort^18^. These patients without PARDS served as a critically ill comparator group whose disease processes were not suggestive of acute lung injury at enrollment.

The present study used a de-identified dataset and blood samples for analysis and was deemed IRB exempt (UCLA IRB #22–001375). Plasma was analyzed from the first collection after enrollment in the study.

### Definitions

We collected demographics (age and sex), comorbidities (presence of chronic pulmonary conditions, neurologic disease, immunocompromise [including oncologic disease or hematopoietic cell transplantation]), elements specific to PARDS (risk factors, chest imaging, and oxygen saturations), illness severity (pediatric risk of mortality score [PRISM III]).

Vital sign, ventilator, and laboratory data were used to calculate organ dysfunction indices (oxygen saturation index [OSI]; pediatric logistic organ dysfunction score[PELOD]−2). Daily OSI^19^ (index of pulmonary dysfunction) and PELOD-2^20^ (index of multiple organ dysfunction) were calculated using the most abnormal values from each calendar day. As a sensitivity analysis, we excluded the respiratory component of the PELOD-2 scoring system to derive the non-pulmonary PELOD-2 score. Data collection focused on the first week of IMV (day 0 = day of intubation) with organ dysfunction scores recorded once daily up to 7 days unless the patient died or was discharged from the PICU (whichever occurred first). We calculated maximum organ dysfunction and cumulative (∑) organ dysfunction scores during this timeframe for use in regression analyses. Given that plasma collection could have been contemporaneous to (or occurred after) maximum organ dysfunction, cumulative organ dysfunction scores were computed to preserve exposure-outcome temporal alignment and provide a more complete measure of organ dysfunction burden.

Clinically relevant outcomes included 28-day ventilator free-days and PICU-free days. Ventilator-free and PICU-free days were determined by subtracting total ventilator or PICU days from 28 in survivors. Patients with ≥ 28 days of IMV, PICU admission, and non-survivors were assigned a value of 0.

### Molecular measurements

#### Glycosaminoglycans (GAGs)

**Fig. 1:**
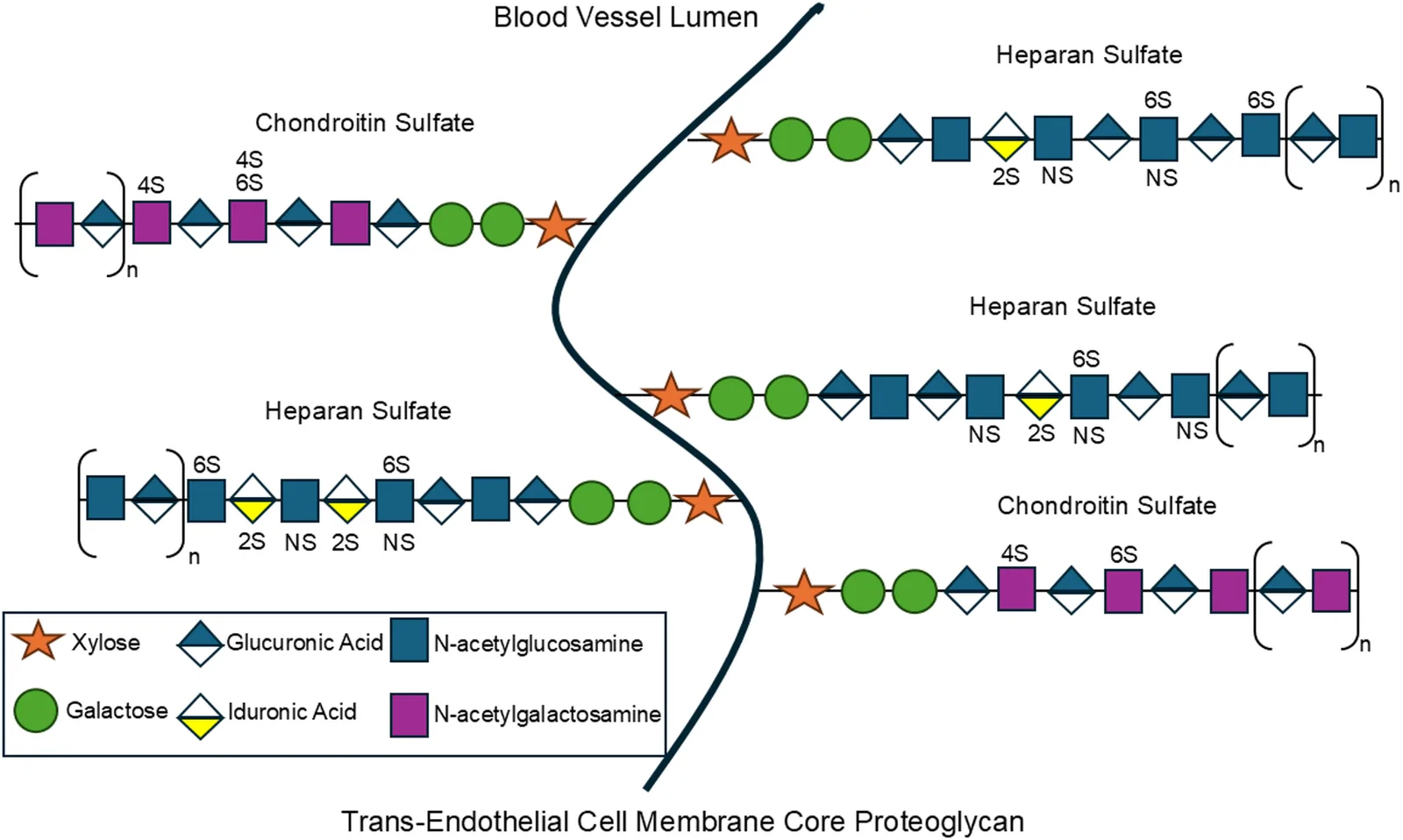
The endothelial glycocalyx. The main constituents include proteoglycans (e.g., syndecan-1) and glycosaminoglycans (GAGs), such as heparan sulfate (HS), chondroitin sulfate (CS), and hyaluronan (HA). GAGs are linear polysaccharides composed of repeating disaccharide units consisting of a hexuronic acid (glucuronic acid [GlcA] or iduronic acid [IdoA]) linked to a hexosamine (N-acetylglucosamine [GlcNAc] or galactosamine [GalNAc]). HS and CS can be further modified by sulfation of their disaccharide units. HS chains typically consist of 50 to 250 disaccharide units (20–100 kDa) and exhibit staggering structural complexity due to variable sulfation at the 6-*O-* (“6S”) and/or amino (“NS”) positions of GlcNAc, as well as the 2-*O-* (“2S”) position of IdoA. This pattern of sulfation along the HS chain generates a heterogeneous distribution of negative charge, enabling highly specific interactions with positively charged regions of soluble and cell-surface proteins. In contrast, HA (not pictured) is non-sulfated and, unlike other GAGs, is not covalently attached to the endothelial cell surface via proteoglycans. Instead, it associates non-covalently with cell-surface glycoproteins (not pictured). Note: As shown in the figure, CS and HS are composed of repeating disaccharide units that can be either sulfated or non-sulfated. The illustration is not drawn to scale and does not depict a fully mature polysaccharide chain. Sulfation at the 3-*O-* (“3S”) position of the GlcNAc residue of HS may also exist within glycocalyx HS, but glyco-analytical techniques at the time of analysis were insensitive to detection of this motif.

We employed a state-of-the-art mass spectrometry-based glycomic approach to examine eGCX damage in children with and without PARDS through plasma GAG profiling. This method enables simultaneous quantification (ng/mL) and structural characterization of HS, chondroitin sulfate (CS), and hyaluronan (HA) polysaccharides following their enzymatic depolymerization into constituent disaccharides. HS consists of repeating disaccharides of uronic acid (ΔUA) linked to *N*-acetylglucosamine (GlcNAc) or *N*-sulfated glucosamine (GlcNS); CS comprises ΔUA linked to *N*-acetylgalactosamine (GalNAc); and HA (which is non-sulfated) comprises ΔUA linked to GlcNAc. This technique further enables detailed GAG structural characterization, including the presence and pattern of HS and CS disaccharide sulfation. A schematic of the eGCX and its GAG constituents is shown in Fig. 1.

In brief, GAGs were isolated, desalted, and enzymatically depolymerized into disaccharide units using recombinant heparin lyases I, II, and III and chondroitin lyase ABC, followed by derivatization with 2-aminoacridone (AMAC). A mixture containing ^13^C-labeled disaccharide internal standards^21^ was similarly AMAC-labeled. After derivatization, samples and standards underwent C18 solid-phase extraction (SPE) to remove dimethyl sulfoxide (DMSO). The resulting disaccharides were measured using high performance liquid chromatography tandem mass spectrometry (HPLC-MS/MS).

This approach yields up to 17 independently quantified disaccharides: eight HS disaccharides (0S [non-sulfated], 2S, 6S, NS, 6SNS, 2SNS, 2S6S, TriS [2S6SNS]), eight CS disaccharides (0S [non-sulfated], 2S, 6S, 4S, 2S6S, 2S4S, 4S6S, TriS [2S4S6S]), and one HA disaccharide. Total (unfractionated) HS and CS represent a sum of their respective disaccharides. Disaccharides with > 20% of values below the limit of detection (LOD), reflecting substantial left-censoring, were excluded rather than imputed to avoid bias, resulting in 12 disaccharides (7 HS, 4 CS, and 1 HA) retained for downstream analyses. Further methodological details are provided in prior publications^22–24^ and the e-supplement.

#### Proteins and proteoglycans

Protein biomarkers previously studied in ARDS and associated with innate immune activation, endothelial activation, and lung epithelial injury were measured in plasma alongside proteoglycans, endocan and syndecan-1 (a main HS proteoglycan), using two magnetic bead multiplex panels (Luminex, R&D Systems, Minneapolis, MN): a 5-plex high performance, high sensitivity human cytokine panel and a 11-plex human discovery assay (e-supplement).

#### HPSE activity

The enzymatic activity of HPSE (U/mL) was measured using 20 µL of human plasma in a total reaction volume of 100 µL using a HPLC-MS/MS-based method developed by our group^25^. The assay enables quantitative measurement of HPSE activity in complex biological matrices, including plasma, and demonstrates strong linearity (R^2^ = 0.99), high sensitivity (LOD = 3.74 ng/mL), and resistance to interference from heparanase-2 (HPSE-2). The reaction mixture consisted of 20 µL plasma, 0.2 nM dekaparin, and 73 µL enzymatic buffer (50 mM sodium acetate, pH 5.0, and 0.15 mM sodium chloride). Samples were incubated at 37 °C for 1 h, after which enzymatic activity was terminated by heat denaturation. A ^13^C-labeled GlcNS6S-glucuronic acid (GlcA) recovery calibrant was added as an internal standard, and the mixture was passed through a 30 kDa molecular weight cut-off centrifugal filter (Amicon^®^ Ultra-0.5) to separate disaccharides from plasma proteins. The filtrate was vacuum-dried and reconstituted in double-distilled water. Samples were then analyzed by HPLC-MS/MS to quantify GlcNS6S-GlcA levels (ng/mL) relative to the ^13^C-labeled internal standard, enabling direct correlation between disaccharide concentration and HPSE enzymatic activity, where one unit of HPSE was defined as the amount of enzyme that generates 1 ng of disaccharide product from dekaparin substrate per hour. The HPSE activity assay utilizes a specific HS disaccharide readout that is not common to native HS and is thus not impacted by endogenous circulating HS levels.

### Statistical analysis

Full methodological details are provided in the e-supplement, with all studied biomarkers listed in e-Table 1. The primary analyses are summarized below.

Principal component analysis (PCA) is an unsupervised dimensionality reduction technique that reduces a multidimensional set of variables into a smaller number of orthogonal components to identify the dominant patterns of variation. As an unsupervised approach, PCA does not require a priori assumptions regarding relationships among variables^26^. In the present study, PCA was used to identify patterns of inter-patient biological variation across GAG, proteoglycan, and protein biomarkers and to determine which biomarkers contributed most strongly to those patterns. PCA was performed on the correlation matrix of natural log-transformed and standardized biomarkers (*n* = 28) using the *prcomp* function (R version 4.2.1, R Foundation for Statistical Computing, Vienna, Austria). Principal components (PCs) were retained based on scree plot inspection and permutation testing (1,000 iterations) to identify PCs explaining more variance than expected by chance and were interpreted as candidate biological axes.

Each PC represented a weighted linear combination of the measured biomarkers. Loading coefficients quantified the magnitude and direction of each biomarker’s contribution, with larger absolute loadings indicating greater influence on the corresponding PC. Loadings were visualized using heatmaps and biplots, and biomarkers with the largest absolute loadings were interpreted as the primary contributors to each PC.

To evaluate the clinical relevance of these biological patterns at the patient level, PC scores were calculated for each participant. These unitless scores reflect the extent to which an individual’s biomarker profile aligns with a given PC and represent patient-level summaries of the dominant patterns identified by the analysis. In children with PARDS, PC scores were subsequently evaluated for associations with organ dysfunction and clinical outcomes using multivariable linear regression. Standardized regression coefficients (β) with 95% confidence intervals (CIs) were estimated, where β represented the change in the outcome (in standard deviation [SD] units) associated with a 1-SD increase in PC score.

We next examined whether structural features of HS, specifically the relative composition of sulfated versus non-sulfated (HS-0S) disaccharides, differed between children with and without PARDS. A composite sulfated HS metric was calculated as the sum of concentrations (ng/mL) of sulfated HS disaccharides (2S, 6S, NS, 6SNS, 2SNS, TriS), and the proportion of sulfated HS was defined as this sum divided by total (unfractionated) HS. We then used linear regression to model the proportion of sulfated HS as a function of log-transformed total HS. Effect modification by PARDS status was assessed by including an interaction term in the regression model (log total HS * PARDS status), with between-group differences in slopes (Δ slope) estimated with 95% confidence intervals. Analyses were repeated for CS.

Exploratory analyses examined associations between HPSE activity and circulating total HS and respective HS disaccharides using Spearman correlation (ρ) and regression models. In a prior study^27^, we identified three distinct PARDS patient clusters (Cluster 1, *n* = 12; Cluster 2, *n* = 19; Cluster 3, *n* = 5) characterized by stepwise increases in absolute concentrations of all HS disaccharides. Cluster 3 identified a severe eGCX shedding phenotype associated with higher disease severity and worse clinical outcomes. We therefore examined whether the relative proportion of sulfated HS and HPSE activity differed across these previously defined PARDS clusters.

Finally, given the modest sample size, prespecified sensitivity analyses accounting for potential influential observations were performed to assess the robustness and internal validity of the findings (e-supplement).

## Results

### Patient characteristics

This study included 46 children: 36 children with PARDS and 10 without PARDS. Demographic characteristics, clinical variables, illness severity were similar between groups (e-Table 2). Plasma collection occurred a similar number of days after initiation of IMV in patients with and without PARDS (median 4.6 days; interquartile range [IQR] 3.4–5.6 vs. 4.3; IQR 3.1–5.3, respectively). OSI was higher in children with PARDS (8.3; IQR 5.4–13 vs. 1.9; IQR 1.8–4.4).

### Principal component analysis of circulating GAGs, proteoglycans, and proteins

**Fig. 2:**
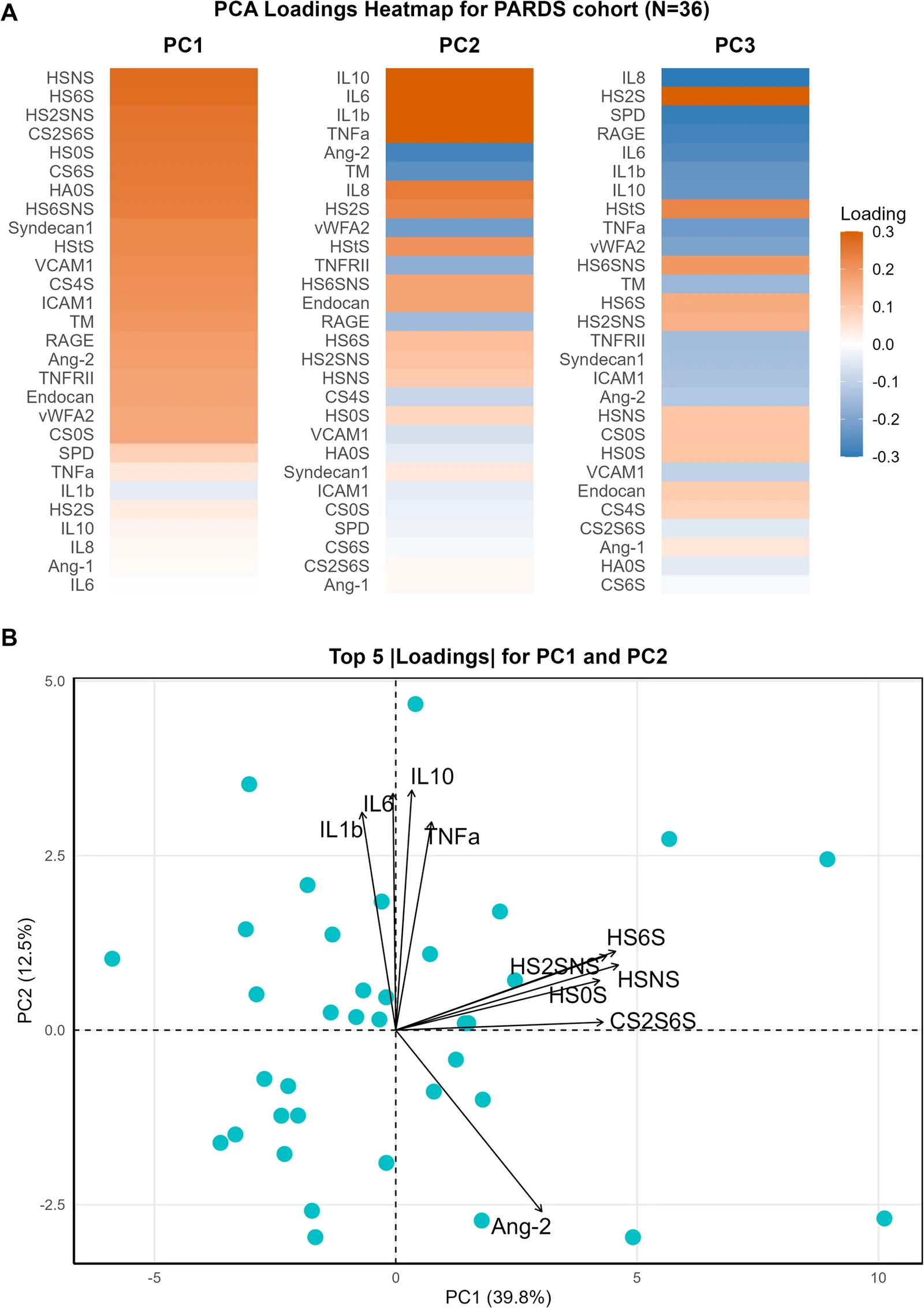
(**A**) Principal Component Analysis (PCA) Loadings Heatmap (PARDS cohort; *N* = 36). Each principal component (PC) represents an orthogonal axis of variance and is a weighted linear combination of the 28 biomarkers. The heatmap displays biomarker loading coefficients (rows) for each PC1-PC3 (columns). Biomarkers are ordered within each panel by descending |loading| to highlight those contributing most strongly to each component (variables contributing most strongly to each component are shown at the top of each panel). Color intensity reflects the magnitude and direction of the loading coefficient, with blue indicating negative loadings, white near zero, and orange indicating positive loadings. Variables with positive loadings are positively correlated with a given PC. (**B**) PCA Biplot (PARDS cohort). The biplot illustrates the distribution of patients along PC1 (39.8% of total variance) and PC2 (12.5% of total variance). Arrows represent biomarker loadings; longer arrows indicate stronger contributions, and their direction reflects the relationship of each biomarker with a given PC. For clarity of data presentation, only the top five biomarkers with the largest |loading| on PC1 and PC2 are displayed. Each point represents a patient, positioned based on their biomarker profile across PC1 and PC2. Points that are closer together indicate more similar biomarker profiles. Points oriented in the direction of an arrow are associated with higher values of the corresponding biomarker. PARDS = Pediatric Acute Respiratory Distress Syndrome.

In children with PARDS, PC1-PC3 were retained based on scree plots and permutation testing (e-Fig. 1), together explaining 63% of the total variance (PC1: 40%, PC2: 12%, PC3: 11%; all *p* < 0.0001). PC1, the dominant axis of biological variation, was characterized by an endothelial-associated signature, with eGCX constituents, particularly HS disaccharides (HS-NS, HS-6S, HS-2SNS, HS-0S) and the CS disaccharide CS-2S6S, exhibiting among the largest positive loading coefficients. In contrast, PC2 was defined by high positive loadings of inflammatory markers, including interleukin (IL)−1β, IL-6, IL-10, and TNF-α (Fig. 2A). After exclusion of identified influential observations that included two patients from Cluster 3 (e-Fig. 2A), HS disaccharides modified with 6-*O*- and *N*-sulfation remained the primary contributors to PC1 (e-Fig. 2B). Bootstrap resampling demonstrated that the magnitude and direction of loadings for each PC were preserved across resampled datasets, supporting the robustness of the identified PCA structure (e-Table 3). The PCA biplot PC1 and PC2 is shown in Fig. 2B.

**Fig. 3:**
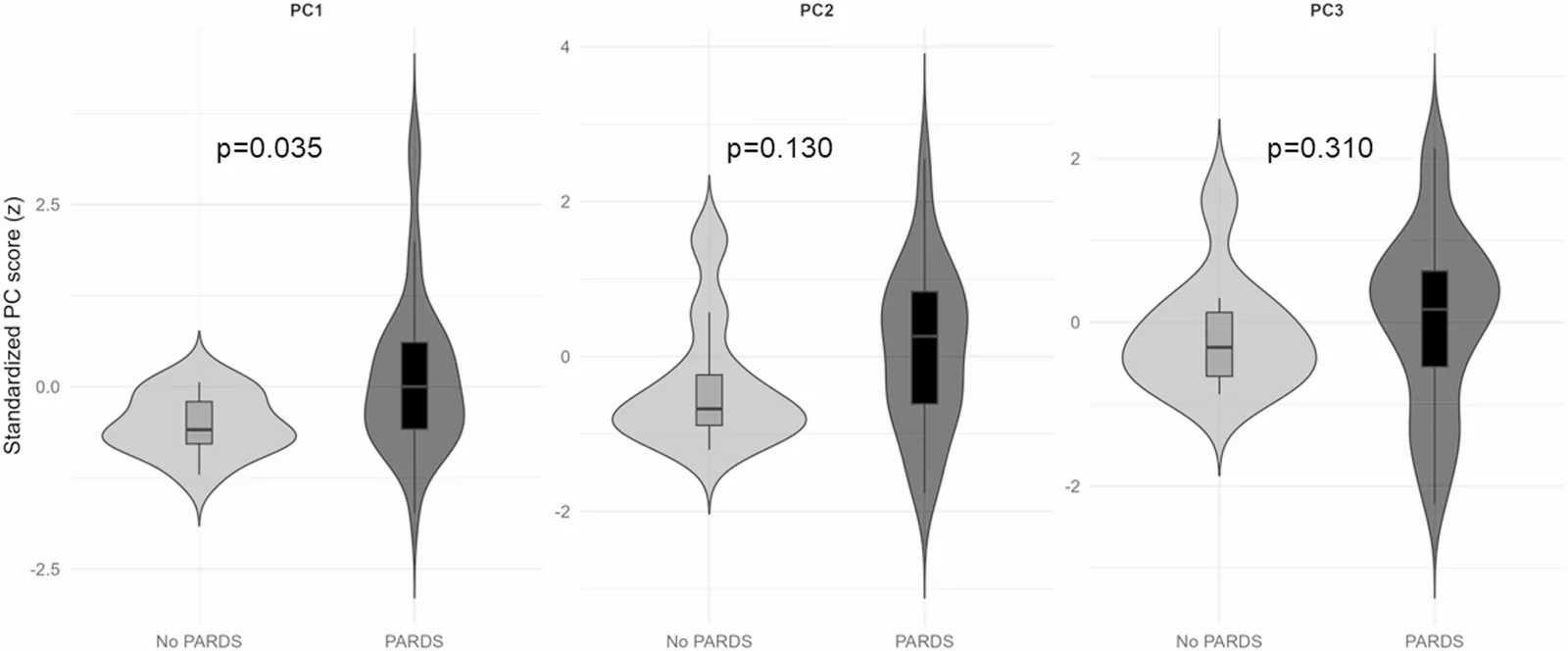
Standardized PC scores by PARDS status. PC scores (unitless values reflecting the alignment between an individual’s biomarker profile and each component) were calculated for each patient. PC1 scores were significantly higher in children with PARDS compared to those without, whereas PC2 and PC3 scores did not differ significantly between groups. *p* < 0.05 was statistically significant. PC = principal component; PARDS = pediatric acute respiratory distress syndrome.

Incorporation of children without PARDS into the PCA yielded similar PC axes (variance PC1: 38%, PC2: 13%, PC3: 10%; all *p* < 0.0001) with preserved loading coefficients (e-Fig. 3A; e-Table 4). PARDS and non-PARDS patients visually separated along the PC1 and PC2 axes (e-Fig. 3B), with significantly higher PC1 scores (reflecting higher values of positively loaded PC1 variables consistent with greater eGCX shedding and endothelial activation) observed in children with PARDS (*p* = 0.035; Fig. 3).

Among children with PARDS, higher PC1 scores were associated with higher ∑OSI, ∑PELOD-2, and ∑non-pulmonary PELOD-2 controlling for PC2 and PC3 (Table 1). An inverse association between PC1 and 28-day ventilator-free days was observed after adjustment for PC2 and PC3 (e-Table 5).

**Table 1:** Association between PCs and max and cumulative organ dysfunction indices.

Principal component	Max OSI β (95% CI)	∑OSI β (95% CI)
PC1	0.13 (95% CI −0.20, 0.46), *p* = 0.437	0.52 (95% CI 0.22, 0.81), *p* = 0.001
PC2	0.25 (95% CI −0.08, 0.59), *p* = 0.133	0.03 (95% CI −0.26, 0.32), *p* = 0.855
PC3	0.19 (95% CI −0.14, 0.53), *p* = 0.246	−0.23 (95% CI −0.52, 0.06), *p* = 0.119
	**Max PELOD-2**	**∑PELOD-2**
PC1	0.34 (95% CI 0.02, 0.66), *p* = 0.035	0.52 (95% CI 0.22, 0.81), *p* = 0.001
PC2	−0.11 (95% CI −0.42, 0.21), *p* = 0.497	0.04 (95% CI −0.25, 0.33), *p* = 0.802
PC3	−0.29 (95% CI −0.61, 0.02), *p* = 0.066	−0.23 (95% CI −0.53, 0.06), *p* = 0.111
	**Max non-pulmonary PELOD-2**	**Non-pulmonary PELOD-2**
PC1	0.29 (95% CI −0.02, 0.62), *p* = 0.071	0.57 (95% CI 0.29, 0.85), *p* < 0.001
PC2	−0.06 (95% CI −0.38, 0.26), *p* = 0.698	0.10 (95% CI −0.17, 0.38), *p* = 0.444
PC3	−0.30 (95% CI −0.62, 0.02), *p* = 0.065	−0.25 (95% CI −0.53, 0.02), *p* = 0.070

Cumulative (∑) organ dysfunction scores were calculated as summative indices up to the first week after initiation of invasive mechanical ventilation. Standardized regression coefficients (β) with 95% confidence intervals (CIs), with β representing the change in the outcome variable in standard deviation units [SD]) associated with a 1 SD change in PC score. Non-pulmonary PELOD has respiratory component removed. *p* < 0.05 was statistically significant. *OSI* oxygen saturation index, *PC* principal component, *PELOD* pediatric logistic organ dysfunction.

### Sulfation patterns of circulating GAGs

Non-sulfated HS (HS-0S) was the predominant disaccharide unit of circulating HS in both absolute and proportional terms in children with and without PARDS (e-Table 6). Although children with PARDS exhibited a higher relative proportion of sulfated HS (and a corresponding lower proportion of HS-0S), the between-group difference in the proportion of sulfated HS was not statistically significant (0.21 [IQR 0.13–0.29] vs. 0.15 [IQR 0.11–0.21]; *p* = 0.130; e-Table 6).

**Fig. 4:**
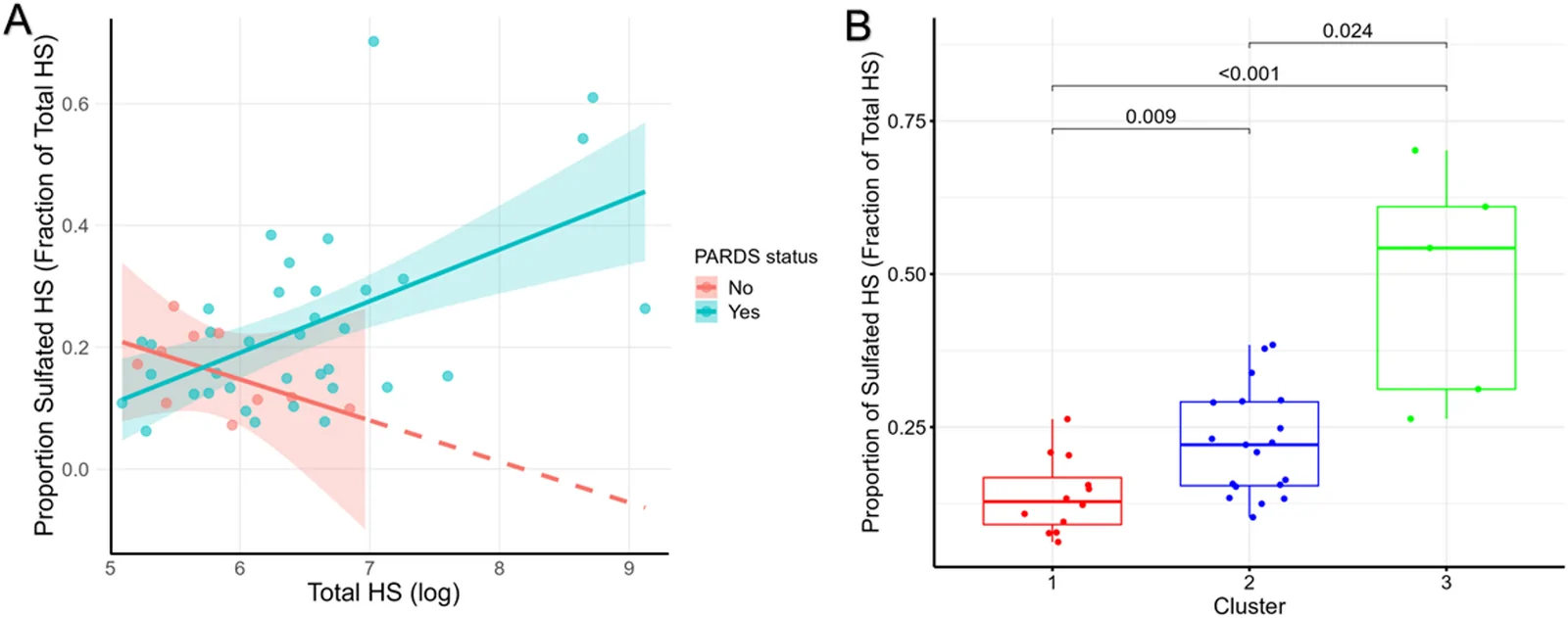
(**A**) The proportion of sulfated HS as a function of total (unfractionated) HS in children with and without PARDS. In pediatric ARDS, higher total HS levels were associated with a higher proportion of sulfated HS (β per log-HS increase = 0.08; 95% CI 0.03, 0.13). In contrast, among ventilated children without PARDS (i.e., those requiring invasive mechanical ventilation but without lung injury or sepsis at enrollment), higher total HS levels were associated with a lower proportion of sulfated HS (β per log-HS increase = −0.06; 95% CI, −0.15, −0.02). A linear model including the interaction term: Proportion Sulfated HS ~ Total HS * PARDS status demonstrated that the slopes differed significantly between groups. *p* < 0.05 for the interaction term was statistically significant. (**B**) The proportion of sulfated HS compared to previously identified PARDS clusters. Each cluster was previously characterized by increasing absolute levels of all HS disaccharides (both sulfated and non-sulfated HS). Cluster 3 was characterized by the highest levels of shedding of eGCX-derived HS and was further associated with greater PARDS severity and worse clinical outcomes. In this figure, the proportion of sulfated HS (the summation of all sulfated HS disaccharides as a fraction of total HS) increased across clusters (Kruskal-Wallis *p* < 0.001), indicating heterogeneity in circulating HS signatures is both quantitative and qualitative, with enrichment of sulfated HS motifs across clusters. Post-hoc Dunn tests identified significant inter-cluster differences (all pairwise *p* < 0.05). *p* < 0.05 was considered statistically significant. HS = heparan sulfate; PARDS = pediatric acute respiratory distress syndrome.

However, when the proportion of sulfated HS was modeled as a function of total (unfractionated) HS, these relationships significantly differed by PARDS status (interaction *p* = 0.020; Fig. 4A). In children with PARDS, the proportion of sulfated HS increased with total HS levels (β per log-HS increase = 0.08; 95% CI: 0.03, 0.13). In contrast, among children without PARDS, increasing total HS levels were associated with a lower proportion of sulfated HS (β per log-HS increase= −0.06; 95% CI: −0.15, −0.02). The relative proportion of sulfated HS differed significantly across PARDS clusters (Kruskal-Wallis *p* < 0.001), with Cluster 3 demonstrating the highest relative proportion of sulfated HS (all pairwise *p* < 0.05; Fig. 4B).

The relationship between total HS levels and the proportion of HS disaccharides expressing 6-*O*- and *N*-sulfation (HS disaccharides that loaded strongly on PC1) also varied by PARDS status (Δ slope = 0.11; 95% CI, 0.05, 0.19; interaction *p* = 0.012, e-Fig. 4A). In addition, the relative proportion of HS expressing these sulfation patterns differed between PARDS clusters (Kruskal-Wallis *p* < 0.001), with the relative proportion of 6-*O*- and *N-*sulfated enriched HS increasing stepwise across clusters (all pairwise *p* < 0.05, e-Fig. 4B).

The proportion of sulfated CS increased with total CS levels among children with PARDS, but there was not strong evidence these relationships varied by PARDS status (data not shown). Sensitivity analyses excluding the same influential observations from Cluster 3 in the PCA models yielded similar effect estimates for all analyses.

### Circulating HPSE activity and correlations with HS

HPSE activity was higher in children with PARDS compared to non-PARDS, although this difference did not reach statistical significance (e-Fig. 5). Children with PARDS exhibited a 10-fold greater variance in HPSE activity relative to non-PARDS (Brown-Forsythe test, *p* = 0.002).

**Fig. 5:**
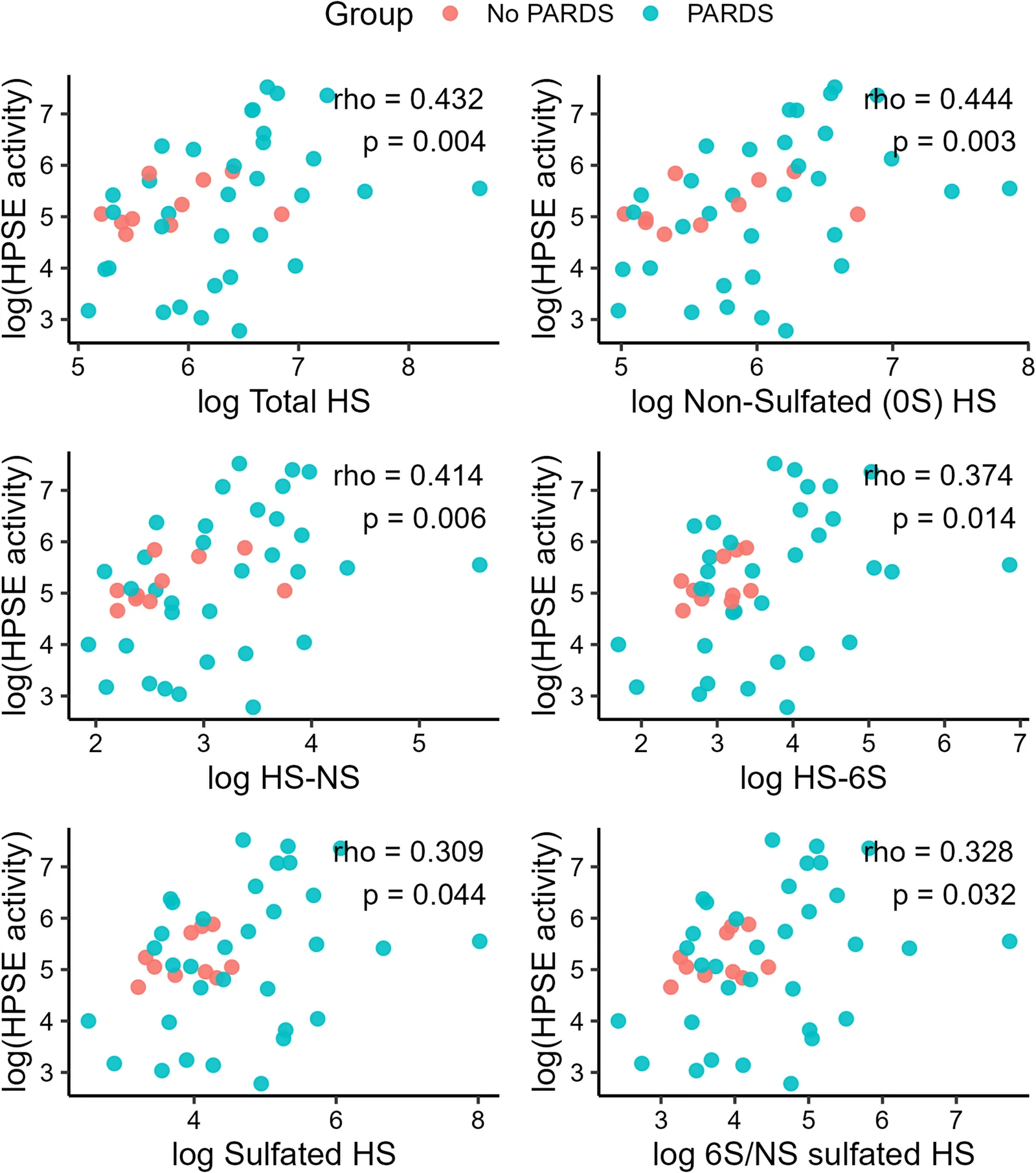
Correlations between circulating HPSE activity and HS. Sulfated HS was defined as the sum of all sulfated HS disaccharides. 6S/NS HS represented the sum of disaccharides enriched in 6-*O*- and *N*-sulfation without 2-*O*-sulfation (HS-6S, HS-NS, and HS-6SNS). HPSE activity was not significantly correlated with HS disaccharides enriched in 2-*O*-sulfation (HS-2S, HS-2SNS, HS-TriS; data not shown) or syndecan-1, an HS proteoglycan (Spearman ρ = 0.29, *p* = 0.057). While our mass spectrometry approach cannot definitively distinguish soluble HS fragments from HS attached to shed proteoglycan ectodomains, these findings suggest that HPSE activity contributes, in part, to the abundance and composition of circulating HS signatures. *p* < 0.05 was statistically significant. HPSE = heparanase-1; HS = heparan sulfate.

Higher HPSE activity demonstrated a modest positive correlation with total HS levels (Spearman ρ = 0.28, *p* = 0.054). Regression diagnostics identified the same two patients from Cluster 3 as influential observations (e-supplement). These patients exhibited the highest total HS levels but among the lowest HPSE activity levels. After excluding these observations, the correlation between HPSE activity and total HS strengthened (ρ = 0.43, *p* = 0.004). HPSE activity also positively correlated with specific HS disaccharides, including HS-0S, HS-NS, and HS-6S. Significant correlations were observed when all sulfated subtypes were analyzed collectively, as well as when 6-*O-* and *N-*sulfated enriched HS were examined in aggregate (Fig. 5).

**Fig. 6:**
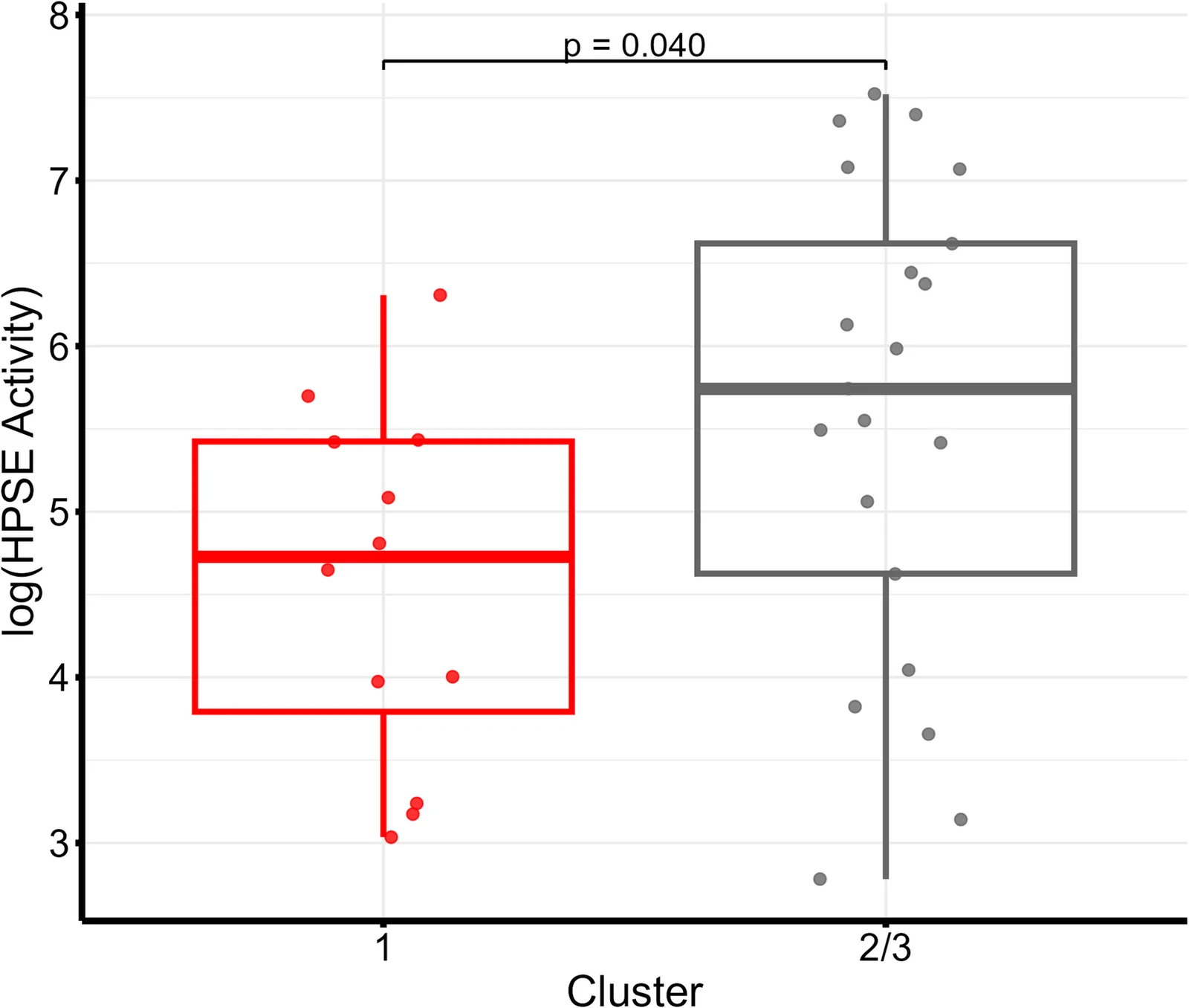
HPSE activity stratified by PARDS clusters. Cluster 1 (*n* = 12) compared to combined Cluster 2 and 3 (*n* = 22) after removal of statistically defined outliers (*n* = 2) from Cluster 3. HPSE activity was significantly higher in children in Cluster 2/3 relative to Cluster 1. *p* < 0.05 was statistically significant. PARDS = pediatric acute respiratory distress syndrome; HPSE = heparanase-1.

Following exclusion of the two influential observations from Cluster 3 and merging Clusters 2 and 3 (due to sample size considerations), HPSE activity was higher in the combined group (*n* = 22) compared with Cluster 1 (*p* = 0.040, Fig. 6).

## Discussion

In this study of mechanically ventilated children with PARDS, we sought to define the biological significance, structural determinants, and potential mechanistic drivers of circulating HS signatures. Our findings provide proof-of-concept that (1) shedding of eGCX-derived HS into the circulation was a distinct and clinically significant contributor to biological heterogeneity; (2) higher total HS levels were associated with selective enrichment of sulfated HS motifs; and (3) in exploratory analyses, HPSE-1 activity positively correlated with circulating HS levels.

Our prior investigations demonstrated that circulating total (unfractionated) HS levels were elevated in children with PARDS compared with those without PARDS^18^. Furthermore, circulating HS profiles were also heterogeneous within PARDS, and children with differentially elevated levels of sulfated and non-sulfated HS disaccharides had worse PARDS severity and clinical outcomes^27^. Building on this prior work, our current findings suggest that release of HS fragments into the circulation (as mechanistic readouts of eGCX shedding) defines a unique topography within the complex biological landscape of PARDS. PCA revealed that HS disaccharides were the main contributors to the dominant pattern of inter-patient biological variation (PC1) within the cohort, exceeding the contributions of other eGCX components (CS and HA) and endothelial activation markers. Importantly, this HS-driven signature was distinct from the inflammatory signature captured by PC2. These findings support the biological inference that eGCX shedding is not merely a correlate or epiphenomenon of systemic inflammation, but instead may reflect a complementary dimension of PARDS biology linked to endothelial injury.

However, not all biological variation is inherently clinically meaningful. PCA enabled evaluation of the clinical relevance of this HS-driven biological signature at the patient level through PC scores. PARDS patients with higher PC1 scores, representing this putative HS-driven endothelial axis, had worse organ dysfunction and clinical outcomes even after adjusting for the inflammatory signature captured by PC2. Studies in adult and pediatric ARDS have demonstrated that the degree of inflammation, characterized by canonical circulating cytokines and markers of innate immune activation, partitions patients into prognostic subphenotypes^28,29^. In contrast, conventional protein biomarkers of endothelial cell activation, although meaningfully altered, have not best distinguished the hyperinflammatory state^30^ or enabled comparable biological subphenotyping. ARDS pathogenesis fundamentally involves disruption of the lung-vascular interface^31^, and the extent and mechanisms of endotheliopathy are also unlikely to be homogeneous across patients. Robust preclinical data implicate eGCX shedding as an early contributor to the endotheliopathy characteristic of ARDS pathogenesis^32,33^. As such, circulating HS signatures may have the mechanistic specificity, temporal resolution, and dimensional depth to identify novel subgroups characterized by distinct endothelial pathobiology.

Although eGCX shedding and innate immune activation emerged as distinct dimensions of biological variation in PARDS, they are likely to be mutually reinforcing in vivo. HS is crucial to establishing chemokine gradients necessary for the directional migration of leukocytes, acting as a ligand for leukocyte selectins, and sequestering cytokines and growth factors, protecting them from enzymatic cleavage^8^. Soluble HS fragments can also signal through MyD88-dependent pathways, including TLR4, leading to NF-κB activation and downstream cytokine release^34^, while inflammatory cytokines, including TNF-α, IL-1β, IL-6, IL-8, and IL-10 reciprocally activate upstream sheddases that contribute to eGCX shedding^35^. Within this framework, innate immune activation and eGCX shedding represent intersecting but non-identical biological axes rather than interchangeable manifestations of the same pathological process. In some patients, convergence of these axes may define a high-risk subphenotype with potential prognostic and therapeutic implications.

Our findings indicate that heterogeneity in circulating HS signatures in PARDS reflects differences not only in abundance but also in structural composition. Although aggregate summaries indicate that circulating HS is predominantly non-sulfated^12,36,37^, these indices overlook how composition varies with the extent of eGCX damage. Notably, in children with PARDS, increasing total circulating HS levels were associated with a disproportionate enrichment of sulfated HS motifs, including those modified with 6-*O-* and *N*-sulfation. In contrast, among children without PARDS, non-sulfated species predominated, a profile perhaps more consistent with non-pathologic or non-enzymatic turnover of the eGCX. Collectively, these findings indicate that measuring HS abundance alone does not fully capture the complexity of eGCX shedding. Instead, enrichment of specific HS sulfation motifs may encode mechanistically and clinically relevant information and facilitate the discovery of novel biomarkers of disease severity in PARDS.

These novel observations are important as preclinical evidence suggests that the HS fragments released from the eGCX are not biologically inert. Rather, fragments of sufficient size and sulfation can influence pathologic cellular signaling through paracrine, autocrine, and endocrine mechanisms^38^. These sulfated HS fragments may function as endogenous danger signals^34,39^, amplifying inflammatory and endothelial signaling, sequestering antimicrobial peptides^40,41^, and further disrupting tissue homeostasis in a sulfation-dependent manner^12^. Prior studies in animal^23^ and human^37^ sepsis show that highly sulfated HS fragments (~ 6–8 saccharides in length) are shed into the plasma, a pattern hypothesized to reflect HPSE-mediated eGCX degradation. HPSE is the only known mammalian endoglycosidase capable of directly cleaving glycocalyx HS. Beyond its direct enzymatic activity on the eGCX, HPSE may modulate eGCX damage indirectly and through non-enzymatic mechanisms^42^. Accordingly, defining the substrate specificity of HPSE and the HS fragments it generates has attracted considerable attention, as these pathways may reveal novel therapeutic targets in critical illness where microvascular endotheliopathy is a common pathway of disease pathogenesis and severity.

Our study provides preliminary evidence that in PARDS, shedding of eGCX-derived HS into the circulation is shaped, in part, by the enzymatically active HPSE. Using a novel functional HPSE assay^25^, we linked the circulating levels of HS to a defined upstream mediator of eGCX shedding. HPSE preferentially cleaves GlcA (without 2-*O*-sulfation)-GlcNS linkages where the GlcNS residue carries 6-*O*- and/or 3-*O*-sulfation, indicating a requirement for defined sulfation motifs within highly sulfated HS domains^43–45^. We observed correlations between HPSE activity and specific HS disaccharides including 6-*O*- and *N*-sulfated species. While it may be tempting to attribute the enrichment of 6-*O*- and *N*-sulfated HS fragments in PARDS solely to HPSE-mediated cleavage, HPSE activity also correlated with non-sulfated HS. Furthermore, HS sulfation is regulated by multiple, interconnected mechanisms, including 6-*O*-sulfatase activity^36^, changes in systemic clearance^46^, and lysosomal processing of HS fragments^47^, all of which may be altered in critical illness. Accordingly, these observations, while novel, should be interpreted as exploratory and hypothesis-generating.

Our findings raise other important biological considerations. The two statistically aberrant observations in Cluster 3, characterized by the highest circulating HS burden (particularly sulfation-enriched HS), paradoxically exhibited among the lowest measurable HPSE activity, suggesting that more complex regulatory mechanisms may be at play. The relationship between HPSE activity and eGCX shedding may be non-linear, such that eGCX shedding reaches a critical threshold at which substrate availability (i.e., cleavable HS on the endothelial surface) becomes limiting, resulting in lower measurable HPSE activity. Alternatively, regulatory feedback mechanisms, possibly mediated by HS fragments themselves, may suppress HPSE activity^48^. Taken together, these findings underscore the need to clarify how HPSE and HS are temporally associated and their relative contributions to organ dysfunction and clinically relevant outcomes.

A few limitations merit consideration. First, this study is limited by a small sample size and cross-sectional biological sampling, which limits inference regarding temporal dynamics and causality. The timing of plasma collection is particularly important in ARDS, where the precise onset of microvascular endotheliopathy (or any meaningful pathobiology) is difficult to ascertain. Although sampling beyond three days after initiation of IMV was restricted by the original study design, prior research suggests that biomarkers measured several days after clinically recognized illness onset may provide greater prognostic value^49^. Therefore, these findings require replication in larger, independent cohorts to confirm the stability of the PCA-derived components, characterize selective patterns of eGCX shedding, and evaluate associations between circulating HS levels, HPSE activity and clinical outcomes in PARDS. Second, eGCX shedding occurs through additional mechanisms, including matrix metalloproteinase (MMP)-mediated cleavage of proteoglycan core proteins such as syndecan-1^50^. HPSE-mediated shedding of HS from syndecan-1 exposes its ectodomain, making it more susceptible to proteolytic cleavage. In addition, HPSE can simultaneously upregulate MMP activity, indirectly enhancing eGCX shedding^51^. These interactions could not be assessed in the present study and warrant further investigation. Third, our glycomic analytical platform, while state-of-the-art, has important limitations. We are unable to localize shedding to specific organs or vascular beds. In addition, we cannot definitively distinguish soluble HS fragments from HS that remains covalently attached to shed proteoglycan ectodomains. Finally, we cannot fully determine HS chain length with comprehensive sulfation mapping, which could help identify fragments generated by HPSE-mediated shedding and provide greater insight into the origins of circulating HS.

## Conclusions

These findings provide proof-of-concept that eGCX-derived HS shedding identifies a distinct endothelial-derived dimension of biological heterogeneity in PARDS, characterized by patterned enrichment of sulfated HS fragments with potential mechanistic and clinical relevance. Given the preliminary nature of this study and the limited sample size, these observations require validation in larger, independent cohorts. If confirmed, eGCX-derived HS signatures and their regulatory pathways may provide a foundation for biologically informed phenotyping, enabling improved risk stratification and the development of targeted therapeutic strategies in PARDS.

## Supplementary Information

Below is the link to the electronic supplementary material.

Supplementary Material 1

## Data Availability

The datasets used and/or analyzed during the current study are available from the corresponding author on reasonable request.

## Abbreviations

ARDS: Acute Respiratory Distress Syndrome
eGCX: Endothelial glycocalyx
HS: Heparan sulfate
PARDS: Pediatric ARDS
HPSE: Heparanase-1
ARF: Acute respiratory failure
IMV: Invasive mechanical ventilation
PALICC-2: Pediatric Acute Lung Injury Consensus Conference-2
PRISM III: Pediatric risk of mortality score
OSI: Oxygen saturation index
PELOD: Pediatric logistic organ dysfunction score
GAG: Glycosaminoglycan
HPLC-MS/MS: High performance liquid chromatography tandem mass spectrometry
CS: Chondroitin sulfate
HA: Hyaluronan
GlcNAc: N-acetylglucosamine
GalNAc: N-acetylgalactosamine
GlcA: Glucuronic acid
IdoA: Iduronic acid
PCA: Principal component analysis
PC: Principal component
CI: Confidence interval
SD: Standard deviation
IQR: Interquartile range
LOD: Limit of Detection
GlcNS: N-sulfated glucosamine
