# Supplementary material for: Plasma heparan sulfate structural variation and phenotypic heterogeneity in pediatric Acute Respiratory Distress Syndrome
Source: Sci Rep. 2026 Jun 26;16:29449. doi: 10.1038/s41598-026-59291-3 (PMC13594105; doi:10.1038/s41598-026-59291-3)
Supplement: Supplementary file 1 [file 41598_2026_59291_MOESM1_ESM.docx]

**Plasma Heparan Sulfate Structural Variation and Phenotypic Heterogeneity in Pediatric Acute Respiratory Distress Syndrome**

*Colin J. Sallee, MD, MS^1^; Aline B. Maddux, MD, MSCS^2^; Joseph A. Hippensteel, MD, MS^3^; Daniela Markovic, MS^4^; Zhangjie Wang, PhD^5^; Jian Liu, PhD^6^; Kaori Oshima, PhD^7^; Robert P. Richter, MD, MS^8^; Andreas Schwingshackl, MD, PhD^1^; Peter M. Mourani, MD^9^; David Elashoff, PhD^4^; Eric P. Schmidt, MD^7^; Anil Sapru, MD, MAS^1^

^1^Department of Pediatrics, Division of Pediatric Critical Care Medicine, David Geffen School of Medicine at University of California Los Angeles and Mattel Children's Hospital, Los Angeles, CA, USA

^2^Department of Pediatrics, Section of Pediatric Critical Care, University of Colorado School of Medicine and Children's Hospital Colorado, Aurora, CO, United States

^3^Department of Medicine, Division of Pulmonary Sciences and Critical Care Medicine, University of Colorado Anschutz Medical Campus, Aurora, CO, USA

^4^Departments of Medicine and Computational Biology, David Geffen School of Medicine at University of California Los Angeles, Los Angeles, CA, USA

^5^Glycan Therapeutics, Raleigh, NC, USA

^6^Division of Chemical Biology and Medicinal Chemistry, Eshelman School of Pharmacy, University of North Carolina at Chapel Hill, Chapel Hill, NC, USA

^7^Department of Medicine, Division of Pulmonary and Critical Care Medicine, Harvard Medical School and Massachusetts General Hospital, Boston, MA, USA

^8^Department of Pediatrics, Division of Pediatric Critical Care Medicine, University of Alabama at Birmingham Heersink School of Medicine, Birmingham, AL, USA

^9^Department of Pediatrics, Division of Pediatric Critical Care Medicine, University of Michigan Medical School, Ann Arbor, MI, USA

*Corresponding Author:

Colin J. Sallee, MD, MS

10833 Le Conte Ave 12-358 MDCC

Los Angeles, CA 90095

csallee@mednet.ucla.edu

**TABLE OF CONTENTS:**

**SUPPLEMENTAL METHODS:**

**Molecular Measurements……………………………………………………………Page 3-4**

**Statistical Analysis…………………………………………………………………...Page 4**

**SUPPLEMENTAL RESULTS:**

**Sensitivity Analyses………………………………………………………………….Page 5**

**e-Figure 1……………………………………………………………………………....Page 6**

**e-Figure 2……………………………………………………………………………....Page 7**

**e-Figure 3……………………………………………………………………………....Page 8**

**e-Figure 4……………………………………………………………………………....Page 9**

**e-Figure 5……………………………………………………………………………....Page 10**

**e-Table 1……………………………………………………………………………......Page 11**

**e-Table 2……………………………………………………………………………......Page 12**

**e-Table 3……………………………………………………………………………......Page 13**

**e-Table 4……………………………………………………………………………......Page 14**

**e-Table 5……………………………………………………………………………......Page 15**

**e-Table 6……………………………………………………………………………......Page 16**

**SUPPLEMENTAL REFERENCES..................................................................................Page 17**

**METHODS:**

**Molecular Measurements:**

Whole blood was collected in an EDTA tube, placed immediately on ice after collection, and centrifuged at 2500 g for 10 min at 4°C to separate plasma. The plasma was aliquoted and stored at -80°C until analysis. Plasma was analyzed from the first collection after enrollment in the study. The full panel of biological markers measured in this study are presented in e-Table 1.

Protein and Proteoglycan Biomarkers.

We measured protein biomarkers associated with innate immune and endothelial activation using two Luminex multiplex panels (Luminex, R&D Systems, Minneapolis, MN): a 5-plex high performance, high sensitivity human cytokine panel and an 11-plex human discovery assay at the Immune Assessment Core (IAC) at UCLA. Measured analytes from plasma include interleukin (IL)-1β, -6, -8, -10, tumor necrosis factor-α (TNF-α), angiopoietin(Ang)-1, -2, endocan, soluble tumor necrosis factor receptor II (TNFRII), soluble intercellular adhesion molecule-1 (ICAM-1), soluble vascular cell adhesion molecule (VCAM-1]), soluble thrombomodulin (TM), von-Willebrand factor (vWF). We included markers of alveolar-epithelial injury surfactant protein D (SPD) and soluble receptor for advanced glycation end products (RAGE) to contrast the innate immune activation endothelial markers. Our selection of biomarkers was guided by prior adult and pediatric ARDS studies, particularly the canonical markers identifying hyper- and hypo-inflammatory subphenotypes.^1-5^ We further measured a commonly employed marker of endothelial glycocalyx (eGCX) shedding in sydecan-1, a main HS proteoglycan.^6^ Each sample underwent duplicate runs with an intra-assay coefficient of variation <5% and a standard curve R^2^ > 95% for each analyte.

Quantification and Structural Characterization of Glycosaminoglycans (GAGs) using High-Performance Liquid Chromatography-Tandem Mass Spectrometry (HPLC-MS/MS).

A subset of circulating heparan sulfate (HS) and chondroitin sulfate (CS) disaccharides fell below the limit of detection (LOD), consistent with prior reports in adults with acute respiratory failure (ARF).^7^ Because principal component analysis (PCA) is sensitive to incomplete data, disaccharides with >20% values below the LOD, reflecting substantial left-censoring, were excluded rather than imputed to avoid introducing bias. At the time of analysis, the HPLC-MS/MS method could not distinguish between CS-2S4S and HS-2S6S disaccharides; these analytes were below the LOD in 11/46 patients (24%) and were therefore excluded. CS-2S, CS-4S6S, and CS-TriS were undetectable across the entire cohort and were similarly removed. For disaccharides with limited values below the LOD, these were imputed as one-half of the minimum observed value (ng/mL) for the corresponding marker (minimum/2). Across the cohort, HS-TriS, HS-2S, and HS-2SNS were below the LOD in 6, 3, and 1 patient(s), respectively. This resulted in 12 disaccharides (7 HS, 4 CS, and 1 HA) included in the final analysis (e-Table 1). Finally, the HPLC-MS/MS method was not sensitive to detection of 3-*O*-sulfation of glucosamine (GlcNAc) residues of HS, a rare modification implicated in the regulation of microvascular thrombosis.^8^ The relative distribution of individual disaccharides observed in our study (e-Table 6) is consistent with prior reports of vascular GAG composition.^7,9^

**Statistical Analysis:**

Categorical variables were summarized with frequency (%) and compared with chi-square or Fisher’s exact tests, where appropriate. Continuous variables were presented using median and interquartile range (IQR) and evaluated with non-parametric rank-order Spearman correlation. Comparison between 2 groups were conducted using nonparametric Wilcoxon rank-sum tests, while comparisons involving >2 groups were analyzed using Kruskal-Wallis tests followed by post-hoc Dunn’s tests for multiple comparisons.

Principal Component Analysis:

Biomarker concentrations were natural (ln(*x*)) log-transformed to address right skewness and standardized (mean = 0, standard deviation = 1) to enable comparisons across assays. Retained principal components (PCs) were considered as candidate biological axes and were based upon a combination of scree plots (eigenvalue >1.0 using the Kaiser-Guttman rule) and permutation testing. To assess the statistical significance of principal components, 1,000 permutation iterations were conducted. For each iteration, values within each variable were randomly permuted independently to disrupt the multivariate correlation structure while preserving the marginal distribution of each variable. PCA was recomputed for each permuted dataset to generate an empirical null distribution of eigenvalues. Observed eigenvalues were compared to their corresponding null distributions, and components were considered statistically significant if the observed eigenvalue exceeded the 95^th^ percentile of the permutation-derived distribution.

Robustness of the PCA structure was evaluated using Mahalanobis distance to assess multivariate outliers and bootstrap resampling to evaluate loading stability.

Mahalanobis distance was calculated in the two-dimensional space defined by PC1 and PC2 to quantify deviation from the sample distribution. This metric incorporates the variance-covariance structure of the PC scores, providing a standardized measure of distance from the multivariate centroid. Observations with squared Mahalanobis distances exceeding the 97.5^th^ percentile of the chi-square distribution (2 degrees of freedom) were flagged as potential multivariate outliers. Bootstrap resampling was performed by repeatedly sampling observations with replacement (1,000 iterations) and recomputing PCA loadings for each resample. To account for sign indeterminacy in PCA, loadings were directionally aligned to the original analysis prior to summarization. Loading stability was assessed by summarizing the distribution of loadings across iterations, including median estimates and empirical 95% confidence intervals (CIs).

Regression Analyses:

Due to the relatively small sample size and to avoid reliance on strict parametric assumptions we employed bootstrap resampling methods, where appropriate, to generate bias-corrected 95% CIs. This approach provides more robust inference under potential distributional uncertainty. In addition, we assessed the influence of individual observations using Cook’s distance (4/n criterion) and difference in beta (DFBETA) statistics (2/√n criterion) to identify points that could disproportionately affect model estimates. Analyses were evaluated both with and without influential observations to ensure the stability of the findings.

**RESULTS:**

Sensitivity Analyses for Principal Component Analysis (PCA); Regression and HPSE analyses:

Across model specifications, two patients previously contained in Cluster 3 in our earlier investigations,^10^ were consistently identified as potentially influential based on Mahalanobis distance criteria for PCA and Cook’s distance and DFBETA criteria for regression analyses. Sensitivity analyses excluding these observations yielded substantively similar results in the PCA analyses and analyses of sulfation enrichment across children with and without PARDS, suggesting that our findings were robust to potential influential data points. However, in exploratory analyses using our novel HPSE activity assay, observed associations were sensitive to their inclusion. These two observations had the highest circulating total HS concentrations, yet exhibited the sixth and eighth lowest HPSE activity in the entire cohort. Indeed, after exclusion of these patients, increasing higher HPSE activity exhibited a robust linear association with total HS levels (β per log-HPSE increase = 0.22, 95% CI 0.10, 0.34, p=0.014).

**e-Figure 1. Scree plot showing the eigenvalues of the principal components (PC) for the PARDS cohort (N=36).** The Kaiser-Guttman criterion was applied to identify components to retain, with the dashed red line indicating the eigenvalue threshold of 1. Permutation testing was then performed to determine whether the variance explained by each PC exceeded that expected by chance. Based on these analyses, PC1-PC3 were retained for further analysis in the main dataset. The results were similar for the entire cohort (PARDS; N=36; No PARDS; N=10).

**e-Figure 2. (A) Mahalanobis distance**. Mahalanobis distance-based outlier detection identified two samples (highlighted in red) as outliers in the two-dimensional principal component (PC) space. **(B) Comparison of PC1 loadings before and after outlier removal.** Figure demonstrates that the loading coefficients remain largely unchanged after outlier removal. In both cases, 6-*O*- and *N*-sulfated HS disaccharides were the primary contributors to PC1.

**e-Figure 3. (A)** **Principal Component Analysis (PCA) Loadings Heatmap (PARDS (N=36) and no PARDS (N=10).** Heatmap of biomarker loadings across the first three principal components (PC1-PC3). Biomarkers are ordered within each panel by descending |loading| to highlight those contributing most strongly to each component. Color intensity reflects the magnitude and direction of the loading, with a diverging scale centered at zero. **(B) PCA Biplot (PARDS and no PARDS).** The biplot illustrates distribution of patients along PC1 (37.9% of total variance) and PC2 (13% of total variance). Arrows represent biomarker loadings; longer arrows indicate stronger contributions, and their direction reflects the relationship of each biomarker with the principal components. For clarity of data presentation, only the top five biomarkers with the largest |loading| on PC1 and PC2 are displayed. Each point represents a patient, positioned based on their biomarker profile across PC1 and PC2. Points that are closer together indicate more similar biomarker profiles. Points oriented in the direction of an arrow are associated with higher values of the corresponding biomarker. PARDS = pediatric acute respiratory distress syndrome.

**e-Figure 4. (A) Proportion of 6-O- and *N*-sulfated HS and total HS by PARDS status.** Among children with PARDS, higher total HS levels were associated with increased proportion of 6-*O-* and *N-*sulfated HS disaccharides whereas the opposite pattern was observed in non-PARDS. A linear model including the interaction term: Proportion 6-*O-* and *N-*sulfated HS ~ Total HS * PARDS status demonstrated that the slopes differed significantly between groups (interaction p=0.012). **(B) Proportion of 6-*O*- and *N*-sulfated HS disaccharides by PARDS clusters.** Proportion of 6-*O-* and *N-*sulfated HS disaccharides increased stepwise across PARDS clusters. These values represent the proportion of total (unfractionated) HS comprised of disaccharides enriched in 6-*O*- and *N*-sulfation without 2-O-sulfation (HS-6S, HS-NS, HS-6SNS). p<0.05 was statistically significant. HS = heparan sulfate; PARDS = pediatric acute respiratory distress syndrome.

**e-Figure 5. HPSE activity in PARDS vs. no PARDS.** HPSE activity was higher in children with PARDS compared with those without PARDS, although this difference did not reach statistical significance. Children with PARDS exhibited more than a 10-fold greater variance in HPSE activity relative to non-PARDS patients (Brown-Forsythe test, p=0.002). HPSE = heparanase-1; PARDS = pediatric acute respiratory distress syndrome.

**e-Table 1: List of Biomarkers**

| **Glycosaminoglycans** | | **Proteoglycans** | **Inflammation** | **Endothelial Activation** | **Epithelial Injury** |
| --- | --- | --- | --- | --- | --- |
| **HS Disaccharides**  TriS_HS_  NS6S_HS_  NS2S_HS_  NS_HS_  *2S6S_HS_  2S_HS_  6S_HS_  0S_HS_ | **HS Disaccharide Structure**  △UA2S(1,4)GlcNS6S  △UA(1,4)GlcNS6S  △UA2S(1,4)GlcNS  △UA(1,4)GlcNS  △UA2S(1,4)GlcNAc6S  △UA2S(1,4)GlcNAc  △UA(1,4)GlcNAc6S  △UA(1,4)GlcNAc | Endocan  Syndecan-1 | IL-1β  IL-6  IL-8  IL-10  TNF-α  TNFR-II | Ang-1  Ang-2  ICAM-1  TM  VCAM-1  vWF | RAGE  SPD |
| **CS Disaccharides**  *TriS_CS_  *2S4S_CS_  2S6S_CS_  *4S6S_CS_  *2S_CS_  4S_CS_  6S_CS_  0S_CS_ | **CS Disaccharide Structure**  △UA2S(1,3)GalNAc4S6S  △UA2S(1,3)GalNAc4S  △UA2S(1,3)GalNAc6S  △UA(1,3)GalNAc4S6S  △UA2S(1,3)GalNAc  △UA(1,3)GalNAc4S  △UA(1,3)GalNAc6S  △UA(1,3)GalNAc |  |  |  |  |
| **HA Disaccharide**  0S_HA_ | **HA Disaccharide Structure**  △UA(1,3)GlcNAc |  |  |  |  |

HS = heparan sulfate; CS = chondroitin sulfate; HA = hyaluronic acid (hyaluronan); UA = uronic acid; GlcNAc = N-acetyl glucosamine; GalNAc = N-acetyl galactosamine; Ang-1 = angiopoietin-1; Ang-2 = angiopoetin-2; ICAM-1= soluble intercellular adhesion molecule-1; IL- = interleukin-; RAGE = soluble receptor for advanced glycation end-products; SPD = surfactant protein D; TM = soluble thrombomodulin; TNF-α = tumor necrosis factor-alpha; TNFRII = soluble tumor necrosis factor receptor II; VCAM, soluble vascular cell adhesion molecule-1; vWF, von Willebrand factor; 0S HS/CS = non-sulfated HS/CS disaccharide. HA is non-sulfated. *These disaccharides had extensive or complete left censoring due to being below the assay limit of detection (LOD) and were therefore excluded from downstream analyses. Each patient had a total of 28 biomarkers included in the analysis.

**e-Table 2: Cohort Characteristics**

| **Variables** | **PARDS (n=36)** | **No PARDS (n=10)** |
| --- | --- | --- |
| **Demographics**  Age, year  Female sex | 9.3 (0.19-13.7)  16 (44.4) | 6.7 (1.9-10.4)  4 (40) |
| **Comorbidities**  Chronic pulmonary disease  Neurologic disease  Immunocompromise | 10 (36)  10 (36)  7 (19.4) | 2 (20)  2 (20)  0 (0) |
| **Timing of plasma collection**  Days from intubation | 4.6 (3.4-5.6) | 4.3 (3.1-5.3) |
| **PARDS Cause**  Direct (e.g., pneumonia)  Indirect (e.g., sepsis)  Unspecified | 23 (63.9)  9 (25.0)  4 (11.1) | NA |
| **Illness Severity and Organ Dysfunction Indices**  PRISM-III  OSI  PELOD-2  Non-pulmonary PELOD-2 | 4 (0-10)  8.3 (5.4-13)  6.9 (2.2-16)  1.1 (0.54-2.8) | 5.5 (0, 11)  1.9 (1.8-4.4)  10.7 (2.2-12.9)  2.8 (0.55-3.5) |
| **Clinical Outcomes**  28-day ventilator-free days  28-day PICU-free days | 20.5 (16.7-22.7)  15.9 (12.4-20.0) | 21.0 (19.1-22.4)  13.7 (12.3-21.3) |

**e-Table 3. Biomarker Loadings for PARDS cohort (n=36)**

| **Biomarker** | **PC1_median** | **PC1_lo** | **PC1_hi** | **Biomarker** | **PC2_median** | **PC2_lo** | **PC2_hi** | **Biomarker** | **PC3_median** | **PC3_lo** | **PC3_hi** |
| --- | --- | --- | --- | --- | --- | --- | --- | --- | --- | --- | --- |
| **HSNS** | 0.268 | 0.186 | 0.302 | **IL10** | 0.278 | -0.183 | 0.506 | **HS2S** | 0.232 | -0.251 | 0.498 |
| **HS6S** | 0.266 | 0.204 | 0.318 | **IL6** | 0.258 | -0.248 | 0.536 | **IL8** | -0.212 | -0.509 | 0.215 |
| **HS2SNS** | 0.255 | 0.173 | 0.309 | **IL1b** | 0.244 | -0.19 | 0.485 | **SPD** | -0.2 | -0.47 | 0.208 |
| **CS2S6S** | 0.247 | 0.125 | 0.304 | **TNFa** | 0.232 | -0.232 | 0.484 | **RAGE** | -0.172 | -0.371 | 0.124 |
| **HS0S** | 0.243 | 0.154 | 0.287 | **Angiopoietin2** | -0.207 | -0.334 | 0.094 | **IL6** | -0.158 | -0.478 | 0.32 |
| **CS6S** | 0.242 | 0.131 | 0.306 | **HS2S** | 0.203 | -0.255 | 0.492 | **HStS** | 0.156 | -0.171 | 0.358 |
| **HS6SNS** | 0.239 | 0.149 | 0.313 | **Thrombomodulin** | -0.187 | -0.34 | 0.118 | **TNFa** | -0.154 | -0.48 | 0.314 |
| **HA0S** | 0.239 | 0.124 | 0.289 | **HStS** | 0.172 | -0.162 | 0.37 | **IL1b** | -0.15 | -0.448 | 0.324 |
| **HStS** | 0.219 | 0.136 | 0.29 | **IL8** | 0.169 | -0.384 | 0.486 | **vWFA2** | -0.143 | -0.389 | 0.222 |
| **Syndecan1** | 0.214 | 0.166 | 0.285 | **vWFA2** | -0.166 | -0.379 | 0.127 | **IL10** | -0.141 | -0.452 | 0.294 |
| **VCAM1** | 0.212 | 0.16 | 0.292 | **TNFRII** | -0.146 | -0.325 | 0.19 | **HS6SNS** | 0.135 | -0.154 | 0.304 |
| **CS4S** | 0.203 | 0.065 | 0.265 | **HS6SNS** | 0.141 | -0.142 | 0.309 | **Thrombomodulin** | -0.108 | -0.333 | 0.212 |
| **ICAM1** | 0.2 | 0.144 | 0.295 | **RAGE** | -0.137 | -0.352 | 0.201 | **TNFRII** | -0.108 | -0.352 | 0.277 |
| **Thrombomodulin** | 0.194 | 0.047 | 0.3 | **HS6S** | 0.1 | -0.123 | 0.241 | **HS6S** | 0.104 | -0.101 | 0.226 |
| **RAGE** | 0.182 | 0.046 | 0.261 | **Endocan** | 0.093 | -0.107 | 0.324 | **HS2SNS** | 0.097 | -0.105 | 0.226 |
| **Angiopoietin2** | 0.179 | 0.051 | 0.27 | **HS2SNS** | 0.092 | -0.133 | 0.232 | **Angiopoietin2** | -0.095 | -0.368 | 0.258 |
| **TNFRII** | 0.166 | 0.088 | 0.268 | **HSNS** | 0.086 | -0.108 | 0.197 | **ICAM1** | -0.093 | -0.286 | 0.182 |
| **Endocan** | 0.165 | 0.096 | 0.25 | **HS0S** | 0.064 | -0.147 | 0.218 | **Syndecan1** | -0.09 | -0.312 | 0.176 |
| **vWFA2** | 0.159 | 0.009 | 0.246 | **VCAM1** | -0.062 | -0.243 | 0.166 | **CS0S** | 0.08 | -0.29 | 0.378 |
| **CS0S** | 0.154 | 0.07 | 0.294 | **CS4S** | -0.06 | -0.314 | 0.216 | **VCAM1** | -0.08 | -0.274 | 0.18 |
| **SPD** | 0.083 | -0.01 | 0.163 | **ICAM1** | -0.037 | -0.25 | 0.226 | **HS0S** | 0.075 | -0.119 | 0.236 |
| **TNFa** | 0.041 | -0.06 | 0.193 | **Syndecan1** | 0.029 | -0.233 | 0.247 | **HSNS** | 0.074 | -0.085 | 0.193 |
| **IL1b** | -0.041 | -0.211 | 0.072 | **HA0S** | -0.028 | -0.175 | 0.101 | **CS4S** | 0.071 | -0.304 | 0.347 |
| **HS2S** | 0.035 | -0.089 | 0.269 | **SPD** | -0.022 | -0.4 | 0.388 | **Endocan** | 0.066 | -0.21 | 0.348 |
| **IL10** | 0.02 | -0.135 | 0.143 | **Angiopoietin1** | -0.016 | -0.318 | 0.303 | **Angiopoietin1** | 0.039 | -0.413 | 0.435 |
| **Angiopoietin1** | 0.009 | -0.092 | 0.079 | **CS0S** | -0.013 | -0.321 | 0.232 | **HA0S** | -0.027 | -0.195 | 0.146 |
| **IL8** | -0.006 | -0.152 | 0.21 | **CS6S** | -0.004 | -0.171 | 0.131 | **CS2S6S** | -0.025 | -0.167 | 0.123 |
| **IL6** | -0.003 | -0.174 | 0.117 | **CS2S6S** | 0.003 | -0.129 | 0.136 | **CS6S** | -0.003 | -0.214 | 0.193 |

Bootstrap-derived median PCA loadings with 95% confidence intervals (PC_lo & PC_hi) for the first three principal components (PC1-PC3). Biomarkers are ordered within each component by descending absolute median loadings.

**e-Table 4. Biomarker Loadings for entire cohort (N=46; N=36 PARDS, N=10 no PARDS).**

| **Biomarker** | **PC1_median** | **PC1_lo** | **PC1_hi** | **Biomarker** | **PC2_median** | **PC2_lo** | **PC2_hi** | **Biomarker** | **PC3_median** | **PC3_lo** | **PC3_hi** |
| --- | --- | --- | --- | --- | --- | --- | --- | --- | --- | --- | --- |
| **HSNS** | 0.276 | 0.203 | 0.306 | **IL6** | 0.307 | -0.09 | 0.533 | **SPD** | -0.223 | -0.454 | 0.141 |
| **HS6S** | 0.272 | 0.209 | 0.32 | **IL10** | 0.286 | -0.076 | 0.511 | **HS2S** | 0.197 | -0.251 | 0.494 |
| **HS2SNS** | 0.261 | 0.179 | 0.313 | **TNFa** | 0.273 | -0.127 | 0.465 | **RAGE** | -0.197 | -0.377 | 0.109 |
| **CS2S6S** | 0.256 | 0.133 | 0.312 | **IL1b** | 0.273 | -0.091 | 0.468 | **TNFRII** | -0.194 | -0.379 | 0.205 |
| **HS0S** | 0.253 | 0.176 | 0.294 | **IL8** | 0.24 | -0.197 | 0.47 | **IL8** | -0.187 | -0.492 | 0.237 |
| **HS6SNS** | 0.243 | 0.162 | 0.317 | **Angiopoietin2** | -0.21 | -0.343 | 0.117 | **CS0S** | 0.167 | -0.252 | 0.47 |
| **CS6S** | 0.239 | 0.133 | 0.309 | **HS2S** | 0.194 | -0.208 | 0.476 | **CS4S** | 0.16 | -0.244 | 0.417 |
| **HA0S** | 0.238 | 0.138 | 0.282 | **vWFA2** | -0.183 | -0.374 | 0.048 | **Angiopoietin2** | -0.155 | -0.362 | 0.19 |
| **Syndecan1** | 0.225 | 0.181 | 0.31 | **HStS** | 0.177 | -0.123 | 0.347 | **HStS** | 0.15 | -0.151 | 0.365 |
| **HStS** | 0.221 | 0.142 | 0.296 | **Thrombomodulin** | -0.171 | -0.325 | 0.116 | **TNFa** | -0.145 | -0.441 | 0.245 |
| **VCAM1** | 0.198 | 0.144 | 0.281 | **HS6SNS** | 0.146 | -0.13 | 0.296 | **Syndecan1** | -0.134 | -0.297 | 0.129 |
| **ICAM1** | 0.195 | 0.133 | 0.308 | **TNFRII** | -0.122 | -0.323 | 0.195 | **IL1b** | -0.134 | -0.429 | 0.311 |
| **Thrombomodulin** | 0.194 | 0.054 | 0.3 | **CS4S** | -0.114 | -0.344 | 0.168 | **HS6SNS** | 0.13 | -0.137 | 0.314 |
| **RAGE** | 0.183 | 0.04 | 0.267 | **HS6S** | 0.099 | -0.124 | 0.23 | **IL6** | -0.129 | -0.447 | 0.297 |
| **CS4S** | 0.182 | 0.038 | 0.257 | **RAGE** | -0.099 | -0.349 | 0.205 | **ICAM1** | -0.127 | -0.327 | 0.173 |
| **Angiopoietin2** | 0.173 | 0.055 | 0.265 | **VCAM1** | -0.088 | -0.25 | 0.12 | **HS2SNS** | 0.112 | -0.071 | 0.239 |
| **TNFRII** | 0.166 | 0.1 | 0.277 | **HS2SNS** | 0.087 | -0.134 | 0.224 | **HS6S** | 0.111 | -0.051 | 0.231 |
| **Endocan** | 0.153 | 0.088 | 0.244 | **CS0S** | -0.079 | -0.388 | 0.195 | **vWFA2** | -0.111 | -0.372 | 0.243 |
| **vWFA2** | 0.146 | -0.008 | 0.243 | **HSNS** | 0.076 | -0.101 | 0.184 | **Thrombomodulin** | -0.11 | -0.329 | 0.168 |
| **SPD** | 0.107 | 0.03 | 0.191 | **Endocan** | 0.074 | -0.173 | 0.296 | **Endocan** | 0.106 | -0.139 | 0.396 |
| **CS0S** | 0.106 | 0.003 | 0.231 | **ICAM1** | -0.064 | -0.295 | 0.173 | **Angiopoietin1** | 0.105 | -0.312 | 0.395 |
| **HS2S** | 0.059 | -0.067 | 0.297 | **HS0S** | 0.058 | -0.117 | 0.186 | **VCAM1** | -0.102 | -0.282 | 0.163 |
| **TNFa** | 0.054 | -0.06 | 0.215 | **Angiopoietin1** | -0.055 | -0.258 | 0.234 | **IL10** | -0.098 | -0.436 | 0.289 |
| **IL1b** | -0.037 | -0.162 | 0.072 | **Syndecan1** | 0.053 | -0.199 | 0.254 | **HSNS** | 0.068 | -0.053 | 0.187 |
| **IL10** | 0.03 | -0.112 | 0.153 | **HA0S** | -0.033 | -0.174 | 0.096 | **HS0S** | 0.068 | -0.091 | 0.206 |
| **Angiopoietin1** | 0.018 | -0.055 | 0.088 | **SPD** | 0.029 | -0.309 | 0.352 | **CS6S** | 0.031 | -0.19 | 0.237 |
| **IL8** | 0.014 | -0.148 | 0.208 | **CS6S** | -0.027 | -0.199 | 0.099 | **HA0S** | -0.012 | -0.186 | 0.184 |
| **IL6** | 0.011 | -0.153 | 0.141 | **CS2S6S** | 0.015 | -0.112 | 0.139 | **CS2S6S** | -0.008 | -0.136 | 0.132 |

Bootstrap-derived median PCA loadings with 95% confidence intervals (PC_lo & PC_hi) for the first three principal components (PC1-PC3). Biomarkers are ordered within each component by descending absolute median loadings.

**e-Table 5: Association between PCs and 28-day VFDs and PFDs.**

| **Principal Component** | **28-day VFD**  **β (95% CI)** | **28-day PFD**  **β (95% CI)** |
| --- | --- | --- |
| PC1 | -0.33 (95% CI -0.64, -0.02), **p=0.037** | -0.29 (95% CI -0.63, 0.04), p=0.088 |
| PC2 | -0.25 (95% CI -0.56, 0.05), p=0.105 | -0.12 (95% CI -0.45, 0.21), p=0.469 |
| PC3 | -0.28 (95% CI -0.59, 0.02), p=0.074 | -0.13 (95% CI -0.47, 0.20), p=0.427 |

Standardized regression coefficients (β) with 95% confidence intervals (CIs), with β representing the change in the response variable in standard deviation units [SD]) associated with a 1 SD change in PC score. p<0.05 was statistically significant. VFD = ventilator-free day; PFD = PICU-free days; PC = principal components.

**e-Table 6: Heparan Sulfate (HS) and Chondroitin Sulfate (CS) Disaccharides Across PARDS versus No PARDS**

**A. Absolute concentrations of HS disaccharides**

|  | HS-2S | HS-6S | HS-NS | HS-2SNS | HS-6SNS | HS-TriS | Sulfated HS | HS-0S |
| --- | --- | --- | --- | --- | --- | --- | --- | --- |
| PARDS  (n=36) | 4.4  (1.5-9.0) | 38  (17-79) | 22  (13-42) | 5.3  (2.2-15) | 22  (9.2-70) | 4.5  (1.2-14) | 113  (48-209) | 455  (281-699) |
| No PARDS  (n=10) | 2.3  (1.2-4.3) | 22  (14-25) | 11  (9.7-17) | 2.1  (1.1-2.4) | 11  (7.2-15) | 0.72  (0.30-1.4) | 57  (34-69) | 243  (183-394) |

Data expressed as median concentration (ng/mL) with interquartile range (IQR). Total sulfated HS = 2S + 6S + NS + NS2S + NS6S +TriS (2S6SNS). HS-0S = non-sulfated HS. Total HS is the sum of all disaccharides and has been shown in prior analyses.

**B. HS disaccharides expressed as a percentage of total HS**

|  | HS-2S | HS-6S | HS-NS | HS-2SNS | HS-6SNS | HS-TriS | Sulfated HS | HS-0S |
| --- | --- | --- | --- | --- | --- | --- | --- | --- |
| PARDS  (n=36) | 0.77  (0.47-1.4) | 7.6  (5.0-9.5) | 4.1  (3.5-4.8) | 0.92  (0.35-0.90) | 4.2  (2.2-7.8) | 0.74  (0.25-1.9) | 21  (13-29) | 79  (71-87) |
| No PARDS  (n=10) | 0.51  (0.33-1.3) | 6.0  (4.6-7.4) | 4.1  (3.6-4.4) | 0.56  (0.45-2.8) | 2.9  (1.4-5.4) | 0.22  (0.09-0.59) | 15  (11-21) | 85  (79-89) |

Data are expressed as the percentage (%) of total (unfractionated) HS represented by each disaccharide, summarized as median (IQR). Fractions were calculated at the patient level to preserve inter-individual variability.

**C. Absolute concentrations of CS disaccharides**

|  | CS-4S | CS-6S | CS-2S6S | Sulfated CS | CS-0S |
| --- | --- | --- | --- | --- | --- |
| PARDS  (n=36) | 10406  (7636-11497) | 267  (196-427) | 3.3  (1.8-8.1) | 10707  (7824-11868) | 2201  (1922-2581) |
| No PARDS  (n=10) | 10791  (8579-11869) | 288  (225-399) | 1.6  (1.0-2.2) | 11308  (8790-12197) | 2675  (2137-3092) |

Data expressed as median concentration (ng/mL) with interquartile range (IQR). Total sulfated CS = 2S + 4S + 6S + 2S6S. CS-0S = non-sulfated CS. Total CS is the sum of all disaccharides and has been shown in prior analyses.

**D. CS disaccharides expressed as a percentage of total CS**

|  | CS-4S | CS-6S | CS-2S6S | Sulfated CS | CS-0S |
| --- | --- | --- | --- | --- | --- |
| PARDS  (n=36) | 78  (75-81) | 2.6  (1.8-3.9) | 0.02  (0.01-0.08) | 81  (0.79-0.84) | 19  (16-21) |
| No PARDS  (n=10) | 75  (75-78) | 2.5  (1.6-2.7) | 0.01  (0.01-0.02) | 0.80  (0.78-0.83) | 0.20  (17-22) |

Data are expressed as the percentage (%) of total (unfractionated) HS represented by each disaccharide, summarized as median (IQR). Fractions were calculated at the patient level to preserve inter-individual variability.

**REFERENCES:**

1.	Calfee CS, Delucchi K, Parsons PE, et al. Subphenotypes in acute respiratory distress syndrome: latent class analysis of data from two randomised controlled trials. *Lancet Respir Med*. Aug 2014;2(8):611-20. doi:10.1016/S2213-2600(14)70097-9

2.	Calfee CS, Janz DR, Bernard GR, et al. Distinct molecular phenotypes of direct vs indirect ARDS in single-center and multicenter studies. *Chest*. Jun 2015;147(6):1539-1548. doi:10.1378/chest.14-2454

3.	Zinter MS, Spicer A, Orwoll BO, et al. Plasma angiopoietin-2 outperforms other markers of endothelial injury in prognosticating pediatric ARDS mortality. *American journal of physiology Lung cellular and molecular physiology*. Feb 2016;310(3):L224-L231. doi:10.1152/ajplung.00336.2015

4.	Yehya N, Zinter MS, Thompson JM, et al. Identification of molecular subphenotypes in two cohorts of paediatric ARDS. *Thorax*. Oct 9 2023;doi:10.1136/thorax-2023-220130

5.	Lim MJ, Whitney JE, Sallee CJ, et al. Plasma Soluble Intercellular Adhesion Molecule-1 Has a Central Role in Biomarker Network Analysis and Is Associated With Poor Outcomes in Two Distinct Pediatric Cohorts of Acute Respiratory Distress Syndrome and Acute Respiratory Failure. *Crit Care Med*. Jul 1 2025;53(7):e1457-e1469. doi:10.1097/CCM.0000000000006719

6.	Murphy LS, Wickersham N, McNeil JB, et al. Endothelial glycocalyx degradation is more severe in patients with non-pulmonary sepsis compared to pulmonary sepsis and associates with risk of ARDS and other organ dysfunction. *Ann Intensive Care*. Oct 06 2017;7(1):102. doi:10.1186/s13613-017-0325-y

7.	Schmidt EP, Li G, Li L, et al. The circulating glycosaminoglycan signature of respiratory failure in critically ill adults. Clinical Trial. *Journal of Biological Chemistry*. Mar 21 2014;289(12):8194-8202. doi:10.1074/jbc.M113.539452

8.	Xu D, Esko JD. Demystifying heparan sulfate-protein interactions. *Annu Rev Biochem*. 2014;83:129-57. doi:10.1146/annurev-biochem-060713-035314

9.	Hippensteel JA, Anderson BJ, Orfila JE, et al. Circulating heparan sulfate fragments mediate septic cognitive dysfunction. *J Clin Invest*. Apr 1 2019;129(4):1779-1784. doi:10.1172/JCI124485

10.	Sallee CJ, Maddux AB, Hippensteel JA, et al. Circulating Heparan Sulfate Profiles in Pediatric Acute Respiratory Distress Syndrome. *Shock*. Oct 1 2024;62(4):496-504. doi:10.1097/SHK.0000000000002421
